# Cocaine-Induced Chronic Bowel Ischemia Manifesting As Small Bowel Obstruction

**DOI:** 10.7759/cureus.58964

**Published:** 2024-04-24

**Authors:** Iraklis Perysinakis, Georgios Saridakis, Miltiadis Giannarakis, Georgia Kritikou, Eelco de Bree

**Affiliations:** 1 Department of Surgical Oncology, University Hospital of Heraklion, Heraklion, GRC; 2 Department of Gastroenterology, University Hospital of Heraklion, Heraklion, GRC

**Keywords:** ischemic enterocolitis, mesenteric ischemia, small bowel obstruction, gastrointestinal complication, cocaine

## Abstract

Cocaine represents one of the most frequently used recreational drugs worldwide. Cocaine-related disorders mostly affect the nervous and cardiovascular system, although gastrointestinal complications are not negligible and sometimes life-threatening. The most common gastrointestinal manifestations of cocaine abuse are ulceration, infarction, perforation, ischemic enterocolitis, and rarely hemorrhage, with mesenteric ischemia being the underlying pathophysiological mechanism. Herein, we report a rare case of cocaine-induced small bowel obstruction in a young female patient, caused by chronic mesenteric ischemia and excessive intestinal wall fibrosis.

## Introduction

The prevalence of illicit drug abuse and drug-related disorders has been continuously rising during the past decade, resembling a global pandemic. According to the World Drug Report 2023 over 296 million people used drugs in 2021, an increase of 23% compared to the previous decade. Accordingly, 39.5 million people suffer from drug use disorders, representing a 45% increase over 10 years [1]. Even though the vast majority of these patients do not seek medical attention for various reasons, the demand for treating drug-related disorders represents a challenge for health systems. Although gastrointestinal complications of recreational drugs are less common than those of the nervous and cardiovascular systems, they may be life threatening [2]. Familiarity with drug-related complications and their manifestations is important for clinicians because these patients are commonly unable to provide a detailed history due to neurocognitive impairment, which also renders clinical evaluation unreliable. Herein, we present the case of a young female patient with chronic bowel ischemia and small bowel obstruction, due to cocaine use, that was surgically treated in our department.

## Case presentation

A 36-year-old female presented to the Emergency Department of our hospital complaining of abdominal pain, constipation, nausea and vomiting. The patient mentioned recurrent episodes of pain for the past six months. Her symptoms were progressively deteriorating resulting in significant food intolerance, severe malnutrition and 40% weight loss. Past medical history was significant for recreational drug use (opioids, cocaine and cannabinoids), drug abuse-related bipolar disorder and chronic hepatitis C.

**Figure 1:**
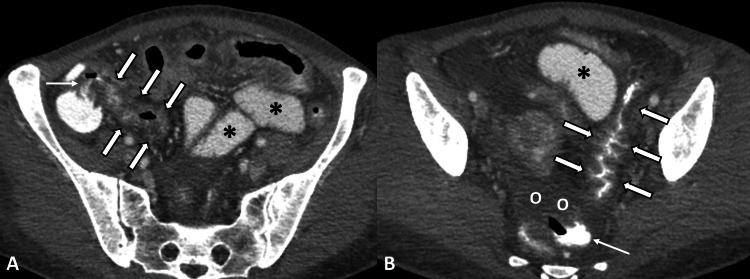
Abdominal CT scan in the portal venous phase (A) Transverse image at the level of the ileocecal valve shows mural thickening of the distal ileum with luminal narrowing (thick arrows), edematous appearance of the iliocecal valve (thin arrow) and marginally dilatated ileal loops (*). (B) Transverse image through the distal colon shows marked wall thickening of the proximal sigmoid colon with effacement of the lumen (thick arrows), dilatated ileal loops and free intraperitoneal fluid (O). Note contrast-filled rectum (thin arrow), indicating non-obstructive ileus.

Upon presentation the patient’s vital signs were within normal limits apart from mild tachycardia. She appeared severely malnourished, weighing only 43kg with Body Mass Index = 17. Clinical examination revealed abdominal distention, reduced bowel sounds and diffuse moderate abdominal tenderness to palpation. Laboratory analysis demonstrated elevated inflammatory markers, thrombocytosis and significant hypoalbuminemia (Table 1). The patient's urine drug screen was positive for cocaine and cannabinoids. An abdominal computed tomography (CT) scan was obtained which showed paralytic ileus and areas of segmental bowel wall thickening, being more prominent at the jejunum, terminal ileum, cecum and sigmoid colon. Several enlarged mesenteric lymph nodes as well as free intraperitoneal fluid were also noted. There were no signs of major visceral artery thrombosis or large-vessel vasculitis (Figures 1A, 1B).

**Table 1:** Laboratory analysis upon presentation WBC, white blood cells; CRP, C-reactive protein; PLT, platelets; Alb, albumin

Laboratory test	Value	Reference range
WBC	14.800/μL	3.800-10.500/μL
CRP	7.7 mg/dl	<0.5mg/dl
PLT	734.000/μL	150.000-450.000/μL
Alb	2.2g/dl	3.5-5.2g/dl

**Figure 2:**
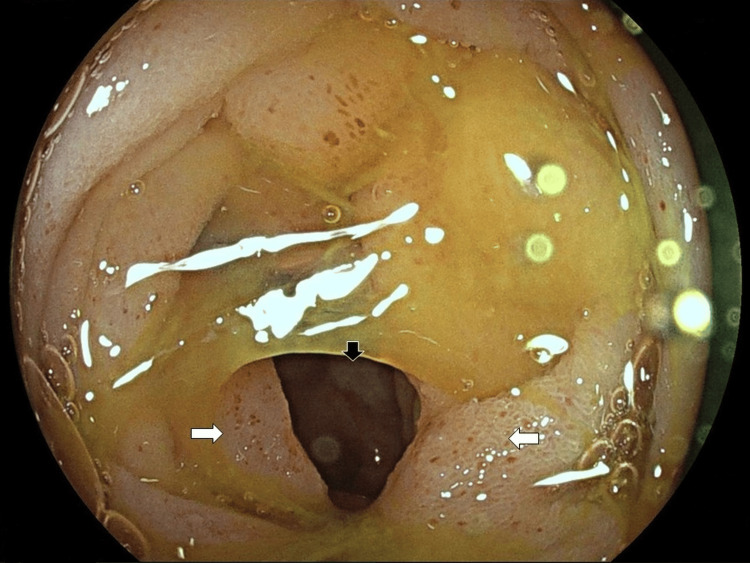
Lower GI endoscopy Lower GI endoscopy in a female patient with history of chronic recreational cocaine use, revealing the presence of ulceration in the terminal ileum (black arrows) and multiple mucosal petechiae (white arrows)

**Figure 3:**
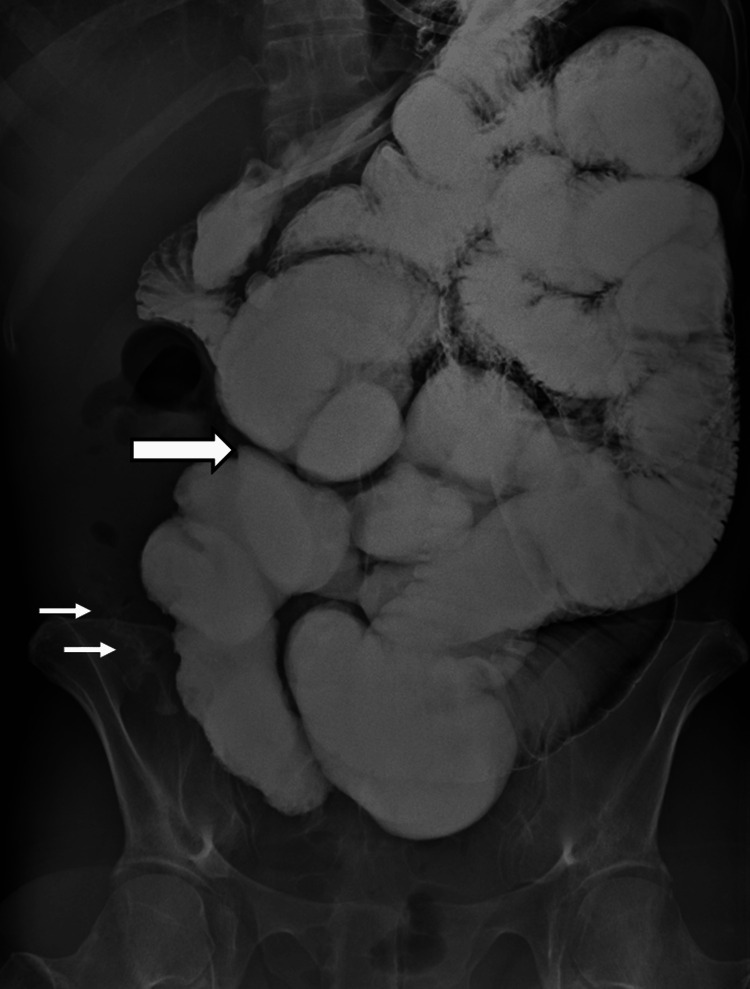
Conventional fluoroscopic enteroclysis Conventional fluoroscopic enteroclysis image depicts dilatation of small bowel loops and a transition point at a distal ileal loop (thick arrow) with minimal passage of contrast material distal to this point (thin arrow), consistent with small bowel obstruction

The patient was admitted to the Gastroenterology Department for further evaluation and treatment. Differential diagnoses included infectious enterocolitis, inflammatory bowel disease, systemic vasculitis and recreational drug-induced enterocolitis. A thorough work-up was undertaken including negative serological tests for systemic vasculitis, negative stool cultures, negative Quantiferon test and negative upper gastrointestinal (GI) endoscopy. Lower GI endoscopy revealed a large ulcer in the terminal ileum with multiple mucosal petechiae and edematous ileocecal valve with erosions (Figure 2). Biopsies from these sites were indicative of bowel ischemia. During hospitalization the patient received empirical antibiotic treatment without response. She did not manage to tolerate oral feeding and was set on parenteral nutrition. Due to progressive deterioration of the patient’s general condition, further workup including an abdominal radiograph and a conventional fluoroscopic enteroclysis, was undertaken and confirmed the presence of small bowel obstruction, with the transition point located approximately 30cm upstream from the ileocecal junction (Figure 3). Consequently, an exploratory laparotomy was decided by a multidisciplinary team meeting.

**Figure 4:**
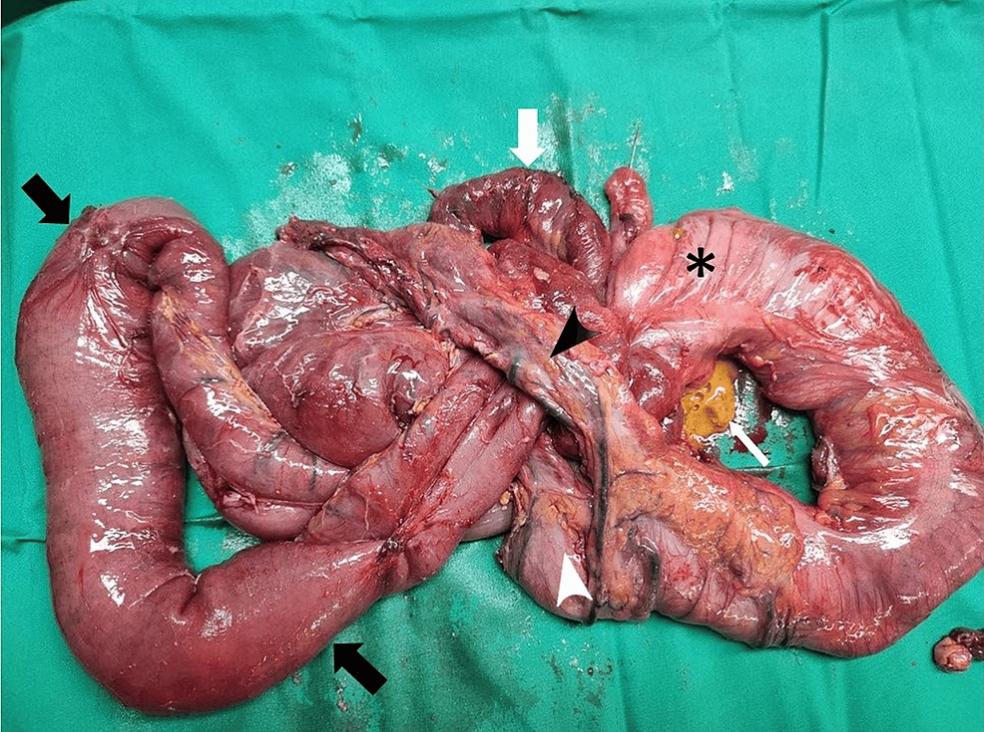
Surgical specimen consisting of the resected distal 100 cm of ileum and the right colon A transition point is evident at the distal ileum between dilated (thick black arrows) and non-dilated (thick white arrow) ileal loops. The presence of dense adhesions at that point has led to the formation of an aggregation of ileal loops, to which the greater omentum (black arrowhead), cecum (asterisk) and transverse colon (white arrowhead) are firmly attached. Note the presence of extraluminal bowel contents (thin white arrow), as a result of unsuccessful efforts to perform adhesiolysis

Intraoperatively hazy intraperitoneal free fluid dilated small bowel loops and extensive bowel adhesions were encountered. No gangrenous or perforated bowel was found. Adhesiolysis was feasible for the first 220cm of the small bowel. From that point downstream the adhesions were extremely dense, forming an aggregation of jejunal loops, so that adhesiolysis was not feasible without causing extensive bowel wall trauma. Provided that the cecum and proximal transverse colon were also firmly attached to the aggregated jejunal loops, en bloc resection of the terminal ileum and right colon was decided (Figure 4). Intestinal continuity was restored by a stapled side-to-side ileotransverse anastomosis, and a drain was placed in the pouch of Douglas. On the third postoperative day, anastomotic leak was identified by intestinal content draining through the abdominal drain. At reoperation complete rupture of the anastomosis was encountered and an end ileostomy and mucous fistula of the transverse colon were formed. Postoperative course was uneventful thereafter. The patient was discharged on the 20th postoperative day, with instructions for enhanced enteral nutrition, dietary supplements, antithrombotic therapy and antidiarrheal medications in order to slow intestinal transit.

**Figure 5:**
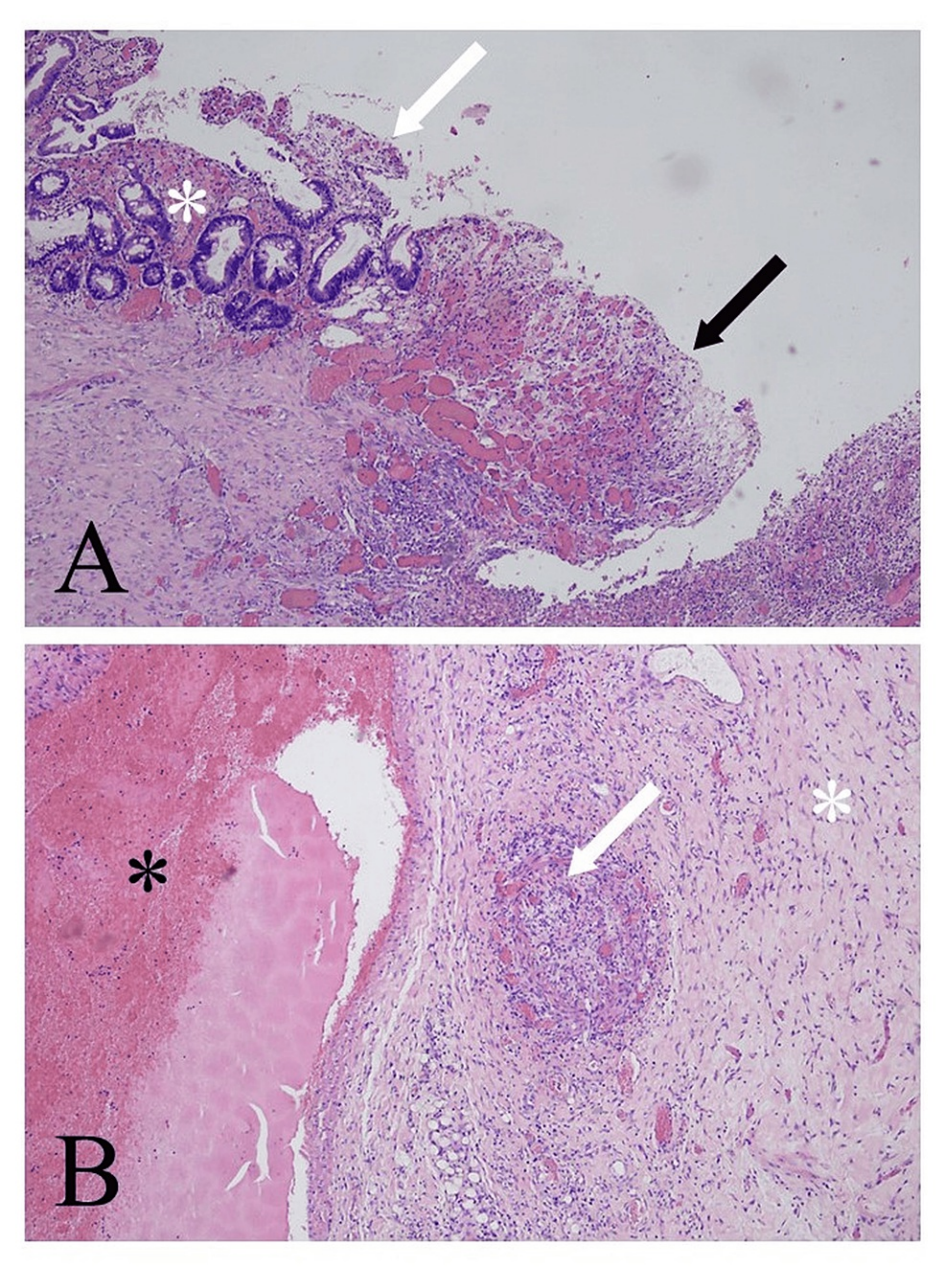
Histopathological picture of cocaine-induced chronic small bowel ischemia (A) Mucosal ulceration (white arrow) with subjacent areas of mucosal regeneration (white asterisk) and inflammatory granular tissue adjacent to the ulcer (black arrow). (B) dilated vessel (black asterisk) with areas of recanalization (white arrow), indicating chronicity. Note the presence of perivascular fibrosis, characterized by deposition of fibrotic tissue around the vessel wall (white asterisk), which is also indicative of chronicity.

Microscopic examination of the surgical specimen revealed the presence of ischemic mucosal ulcers in the small bowel without the evidence of inflammatory bowel disease. Moreover, the absence of inflammation of blood vessel walls ruled out systemic vasculitis as the underlying cause of mucosal ulceration. The ischemic areas were surrounded by excessive accumulation of fibrotic tissue, indicating chronicity. The histopathological picture was compatible with chronic cocaine-induced bowel ischemia (Figures 5A, 5B).

Currently, the patient is regularly followed up by the Nutritional Medicine Department of our Hospital. Six months after hospital discharge, she has made significant progress in terms of her nutritional status and denies continuing cocaine use.

## Discussion

According to the World Drug Report 2023, it is estimated that in 2021, 22 million people had used cocaine [1]. According to the Global Burden of Disease Project, cocaine use disorder was associated with an estimated 3,300 deaths, a rate of 0.8 per 100,000, and 2.6 million disability-adjusted life years in the United States and Canada [3].

The most common gastrointestinal manifestations of cocaine abuse are ulceration, infarction, perforation, and ischemic enterocolitis [4,5]. Rarely cocaine abuse may cause gastrointestinal hemorrhage [6]. Cocaine is a known risk factor for nonocclusive mesenteric ischemia [7]. Cocaine-induced ischemia is mediated by several mechanisms, including vasoconstriction, platelet aggregation, thrombosis, endothelial dysfunction, and finally hindered thrombolysis [2].

In our patient recreational use of cocaine was complicated by chronic intestinal ischemia. The detrimental effects of cocaine on the intestinal microcirculation resulted in ischemic ulcer formation, which in turn led to excessive accumulation of fibrotic tissue on the intestinal wall. The patient suffered recurrent worsening attacks of abdominal pain over a six-month period and finally presented with complete intestinal obstruction at the terminal ileum, which was the most severely affected area.

We carried out a comprehensive search of the literature, using PubMed (http://www.ncbi.nlm.nih.gov/pubmed). The following keywords were used in the search: (cocaine) AND ((intestinal ischemia) OR (bowel ischemia)). Furthermore, a second search was conducted using the keywords: (cocaine) AND ((small bowel obstruction) OR (intestinal obstruction)). No language restrictions were applied.

The initial search resulted in 72 articles for cocaine-induced intestinal ischemia all of which were scrutinized for inclusion. After excluding irrelevant articles and reviews, 39 articles were eligible for critical evaluation. With the exception of two articles, the rest were reports of acute cocaine-related intestinal ischemia, manifesting as gangrenous bowel and/or perforation. Myers et al. reported in 1996 two young patients with chronic mesenteric ischemia caused by thrombosis of large visceral arteries due to cocaine abuse, were treated with visceral revascularization [8]. Hobolth et al. reported in 2009 one case of cocaine-triggered chronic ischemic enteritis of the terminal ileum which was treated conservatively [9].

From the second search, 88 manuscripts for cocaine-associated intestinal obstruction were initially retrieved. Nearly all of them referred to intestinal obstruction in the context of “body packing,” i.e., the practice of swallowing cocaine-filled packets or placing them in body cavities to evade detection by law enforcement. There were only two out of 88 articles regarding intestinal stenosis from cocaine abuse. Sommariva et al. reported one patient with recurrent and reversible episodes of intestinal obstruction, which were attributed to cocaine use [10]. Probably the only similar case to ours was the one reported by Ruiz-Tovar et al. in 2010, in which chronic cocaine-induced ischemic colitis resulted in sigmoid colon stenosis [11].

## Conclusions

Intestinal ischemia, being the second most common gastrointestinal complication of cocaine use, should be always kept in mind in cocaine users presenting with abdominal complaints. Bowel obstruction, although less common than other sequela of cocaine-induced intestinal ischemia, has to be considered in differential diagnosis. Obstruction is generally observed in the colon. To our knowledge, the first case of small bowel obstruction from chronic cocaine abuse, necessitating surgical intervention, is herein reported.
